# Evaluation of Stent Angioplasty in the Treatment of Arteriosclerotic Lesions of the Common Femoral Artery

**DOI:** 10.3390/jcm11102694

**Published:** 2022-05-10

**Authors:** Tanja Böhme, Thomas Zeller, Mohamed Abboud, Ulrich Beschorner, Elias Noory

**Affiliations:** Clinic for Cardiology and Angiology II, University Heart Center Freiburg-Bad Krozingen, Campus Bad Krozingen, Südring 15, 79189 Bad Krozingen, Germany; thomas.zeller@uniklinik-freiburg.de (T.Z.); dr.mohamedabboud@gmail.com (M.A.); ulrich.beschorner@uniklinik-freiburg.de (U.B.); elias.noory@uniklinik-freiburg.de (E.N.)

**Keywords:** peripheral arterial disease, stent angioplasty, common femoral artery

## Abstract

In many vascular segments, endovascular therapy is the treatment of choice for arteriosclerotic lesions. For the treatment of common femoral artery (CFA) lesions, surgical reconstruction is still considered the gold standard. The purpose of this study is to evaluate the safety and efficacy of stent angioplasty for the treatment of common femoral artery (CFA) lesions in a real-world population during a two-year follow up. This retrospective, single-center study includes 250 patients requiring treatment with stent angioplasty of CFA lesions. The primary end point was the target lesion revascularization (TLR) rate. Secondary end points were the overall procedural complication rate, the rate of ipsilateral CFA punctures during follow-up, changes in the Rutherford–Becker class (RBC) and ankle–brachial index (ABI), primary patency rates, amputation rate, time to and the type of TLR. A total of 236 interventions (94.4%) were successfully defined as a residual stenosis < 30%. Periinterventionally, there were 23 complications (9.1%), 3 of which had to be treated surgically. Median follow up was 21 months (average 19.2 ± 7.8). In total, 41 patients (16.4%) needed a TLR. The primary patency rate was 90.8%, 81.2% and 72% at 6, 12 and 24 months, respectively. ABI and RBC were significantly better at all time points compared to baseline. During follow up, seven amputations (three minor and four major) had to be performed. More than half of the patients (56.0%) were punctured at the stented CFA during the follow up. Multivariate logistic regression analysis showed continued nicotine use and coronary heart disease as predictors for TLR. Stent angioplasty for the treatment of CFA lesions is safe and effective. Further studies are needed to compare this endovascular option with surgical therapy.

## 1. Introduction

The incidence of peripheral artery disease (PAD) has increased worldwide [1]. Limitation of pain-free walking distance (intermittent claudication), rest pain or tissue ulceration represent the indications for treatment of PAD [2]. Due to lower invasiveness, endovascular therapy has become the treatment of choice over open surgical therapy in many arterial regions [2,3]. This does not yet include treatment of the common femoral artery (CFA). Here, surgical endarterectomy is still considered the therapeutic “gold standard”. The primary 1-year patency rates after surgical endarterectomy reported in the literature are 85–95% [4]. Numerous small studies indicate that endovascular therapy may have the potential to replace open surgery at least for some anatomical characteristics of CFA lesions [5,6,7,8,9]. In a retrospective study, the use of stents was associated with significant lower 1-year restenosis and target lesion revascularization (TLR) rate and was a protective factor against procedure failure [6]. The TECCO trial [10], a prospective, randomized, multicenter study comparing primary stent angioplasty and open surgical reconstruction for CFA lesions, documented comparable reintervention rates at 2 years. Because of remaining concerns about stent implantation in the CFA, the aim of the present retrospective, monocentric study is to evaluate the technical and clinical outcomes of stent angioplasty in CFA lesions in a real-world patient and lesion population.

## 2. Materials and Methods

Patients treated with atherosclerotic lesions of the CFA between January 2008 and December 2018 were retrospectively selected from a prospective maintained database. The study was approved by the ethics committee of the Albert-Ludwigs University Freiburg, Germany. Approval was given on 2 July 2020. Medical records, angiographies and endovascular procedures as well as duplex ultrasound examinations and ankle–brachial index (ABI) measurements were analyzed. Patients with PAD Rutherford–Becker class (RBC) 2 to 5 with a CFA stenosis ≥ 70% (estimated by duplex ultrasound with a peak systolic velocity ratio of >3.5 and/or visually on angiography) and stent angioplasty of this lesion were included in this analysis. Patients treated with other interventional techniques such as plain balloon angioplasty and atherectomy without final stent placement were excluded. CFA lesions were categorized according to the new classification of Rabellino, et al. [11].

Two-year target lesion revascularization (TLR) rate was the primary end point. Secondary end points were primary patency (restenosis was defined as stenosis with a peak systolic velocity ratio > 2.5 on color-flow duplex ultrasound measuring), overall procedural complication rate, the rate of ipsilateral CFA punctures during follow up, changes in RBC and ankle–brachial index (ABI), amputation rate and predictors on the reintervention rates. The procedural complication rate includes access site complications, target vessel perforation, outflow embolization and compartment syndrome. The time to TLR and the type of TLR (surgery or endovascular procedure) were documented.

According to department standard, follow up visits including physical examination, estimation of the RBC, ABI measurements and duplex ultrasound were scheduled for 6, 12 and 24 months post procedure.

Analyses were performed using SPSS software (version 25.0; SPSS, Chicago, IL, USA). Continuous data are presented as means ± standard deviation; categorical data are given as counts (percentages). TLR-free survival was evaluated using Kaplan–Meier analysis; the survival curves were compared using the Mantel–Cox log-rank test. Univariate logistic regression analysis included the following variables: age, gender, index, smoking status, hypertension, dyslipidemia, diabetes mellitus, initial lesion grade (stenosis versus occlusion), lesion calcification, POBA, DCB or atherectomy use and post procedural residual stenosis. Outcomes of the regression analysis are given as an odds ratio with 95% confident intervals. Significance level was set as *p* < 0.05.

## 3. Results

**Figure 1:**
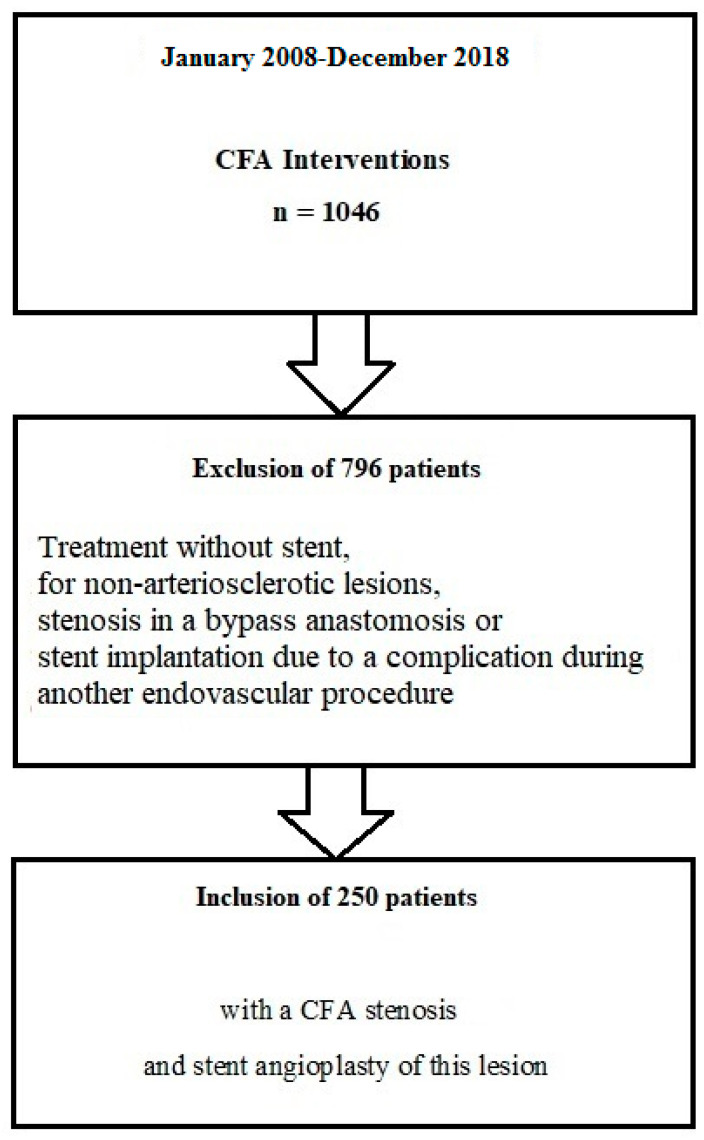
Study flow chart. CFA—common femoral artery.

During the study period, 1046 interventions were performed at the CFA level. According to the inclusion criteria, 250 patients could be included in the analysis. The study flow chart is shown in Figure 1.

Baseline patient characteristics are given in Table 1. The classification of lesions is shown in Table 2. A de novo stenosis was treated in 180 cases (72.0%). In a large number of cases, additional lesions in vessel segments on the ipsilateral or contralateral side were treated. Lesion and interventional details are presented in Table 3. The most frequently implanted stent was the S.M.A.R.T^TM^ stent (Cordis, Miami Lakes, FL, USA). Details of stents used are shown in Table 4.

### 3.1. Acute Outcome

Based on visual estimation, the preinterventional stenosis degree was 86.49 ± 10.7%. A total of 236 interventions (94.4%) were successfully defined as a residual stenosis < 30%. Periinterventionally, there were 23 complications (9.2%), 3 of which had to be treated surgically (2 compartment syndromes, 1 false aneurysm after brachial artery puncture) (Table 5). In 6 of the 10 pseudoaneurysms, no closure device was used.

### 3.2. Follow Up Outcome

**Figure 2:**
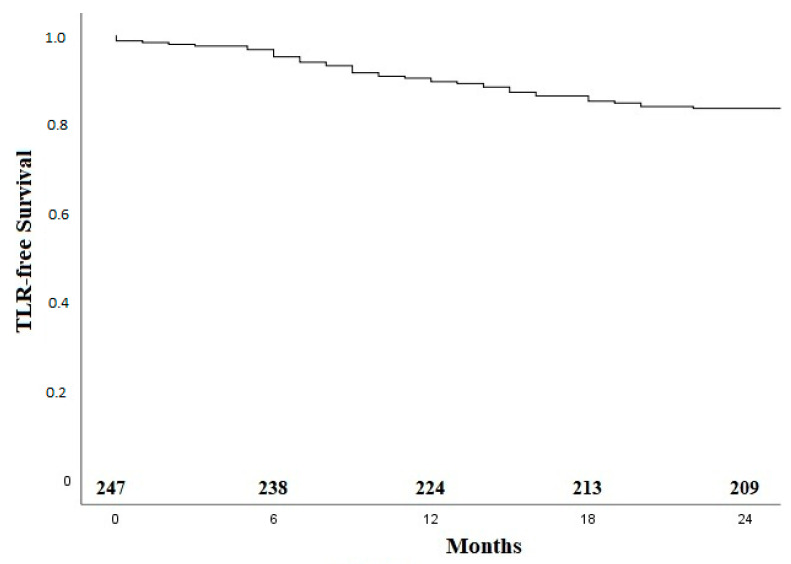
Kaplan–Meier curve for TLR-free survival. TLR—target lesion revascularization.

Median follow up was 21 months (average 19.2 ± 7.8). In total, 41 patients (16.4%) underwent a TLR (Table 6). The survival without TLR by Kaplan–Meier analysis is shown in Figure 2. 

**Figure 3:**
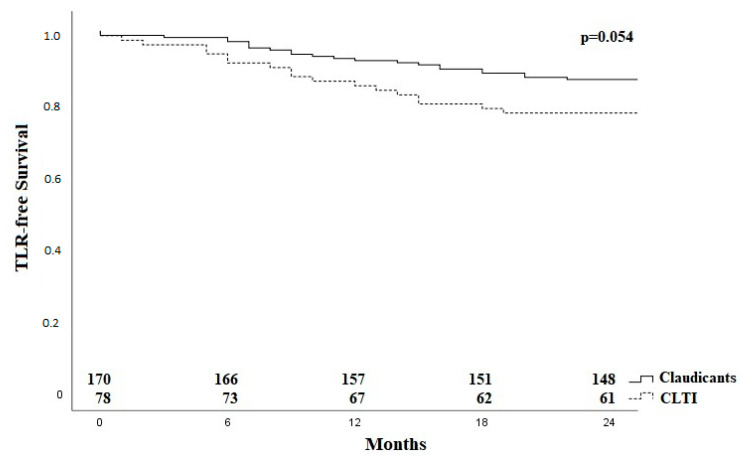
Kaplan–Meier curve for TLR-free survival for claudicants and patients with critical limb ischaemia. CLTI—critical limb ischaemia; TLR—target lesion revascularization.

A total of 26 of the 41 TLRs (63.4%) occurred within the first 12 months. Figure 3 shows the Kaplan–Meier curve regarding TLR-free survival for the group of claudicants and patients with critical limb-threatening ischemia (CLTI).

**Figure 4:**
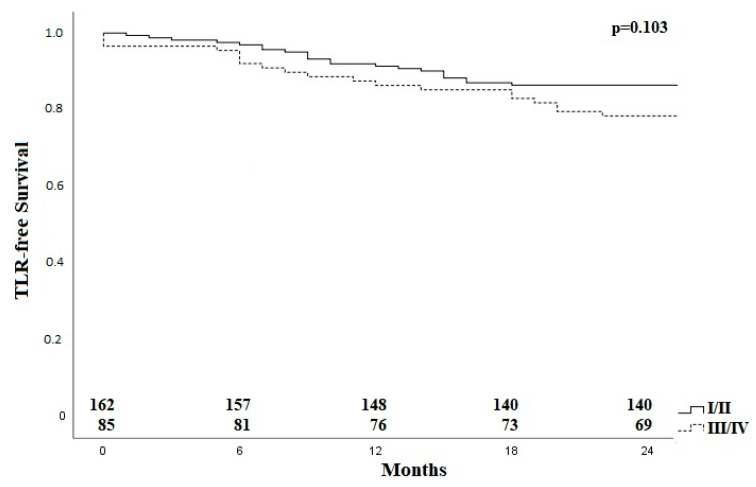
Kaplan–Meier curve for TLR-free survival for patients with lesions with and without bifurcation involvement. TLR—target lesion revascularization, I–IV; classification according to Rabellinio [11].

Applying the Rabellino classification [11], there was no difference regarding TLR after subdivision into group I/II and the groups with bifurcation involvement (III/IV) as shown in Figure 4. 

Duplexsonographically determined the primary patency rate was 90.8%, 81.2% and 72% at 6, 12 and 24 months, respectively. Compared to the baseline, ABI and RBC were significantly better at all time points during follow up (Table 6). During follow up, seven amputations (three minor and four major) had to be performed. Time to amputation ranged from 1 to 14 months (mean 3.4 months) (Table 6).

More than half of the patients (56.0%) were punctured at the stented CFA during the follow up. Access via the stented vessel was most common for a further peripheral angioplasty (*n* = 125, 89.3%). However, access via stented CFA was also used for coronary angiography, endovascular aortic repair or transcatheter aortic valve implantation. Ipsilateral puncture had no significant effect on primary patency (*p* = 0.190) or TLR rates (*p* = 0.058).

Multivariate logistic regression analysis showed continued nicotine abuse and coronary heart disease as predictors for TLR (Table 7).

## 4. Discussion

The CFA is considered a challenging vessel segment for endovascular treatment due to the potential high stress caused by its location in a motion segment. Thus, this vascular segment was traditionally reserved for surgical therapy. However, in recent years, some studies have also shown encouraging results after endovascular therapy, particular the randomized controlled TECCO trial [10]. The present 2-year TLR rate of unselected patients of 16.4% is comparable to both study arms of the TECCO trial (14.4% for the stent cohort and 15.2% for the surgical cohort). However, the proportion of patients with CLTI was lower in the TECCO study than in the present study (30%). In the TECCO study, the proportion was 8% in the surgical group and 18% in the endovascular group. In general, the literature reported TLR rates range from 7 to 20% at 12 months [6,7,8,12,13]. However, in most of these studies, the proportion of patients with CLTI is lower.

In our evaluation, there was no significant difference regarding TLR between the different lesion groups according to Rabellinio et al. [11]; in particular, bifurcation lesions did not result in inferior outcomes as compared to lesions limited to the CFA main stem.

Univariate and multivariate logistic regression analysis showed continued nicotine abuse and coronary heart disease as predictors for TLR. In other studies, age, renal insufficiency, or the presence of CLTI were predictive of reintervention [7,14,15]. Nicotine use has not yet been described as a predictor. On the contrary, one study showed a lower rate of re-intervention in the group of patients smoking 10 or more cigarettes a day [16]. However, this particular study only reports about the course of the disease within the first year after intervention. The presence of coronary artery disease as a predictor for subsequent TLR may be a sign of a more diffuse and progressive kind of atherosclerotic disease in these patients.

The primary patency rate of 81.2% at 12 months is in line with other CFA studies [6,7,8,9]. Slightly higher patency rates are reported after surgical therapy. Kang et al. reported a primary patency rate of 93% at 12 months. Another study found primary patency rates of 97.3% at 6 months and 90.2% at 3 years [4,17].

The complication rate is comparable to those of other studies in this vascular segment [6,8,14] and is driven by access site complications, in particular pseudoaneurysm formation. One pseudoaneurysm of a brachial artery had to be treated surgically; the remaining ones underwent conservative treatment with compression therapy comparable to other studies [6].

At each follow up time point, the present cohort showed persistent significant improvement in ABI and RBC compared to the baseline. This is in line with former trials dealing with endovascular therapy of the CFA [7,18]. One past study showed that the primary sustained clinical improvement was better in patients who underwent stent angioplasty than in patients who underwent angioplasty alone [18]. The major amputation rate of less than 2% is consistent with those of numerous other studies after endovascular therapy of CFA (range 1–3.8%) [6,8,18].

Former concerns that a stent in the CFA would limit future endovascular procedures or surgery were not confirmed in recent studies. Nasr et al. [19] reported unproblematic surgical therapies (aorto–bi–femoral bypasses, iliofemoral bypasses, femoro–popliteal bypasses) involving stented CFA. As in the present work, femoral access was possible for following endovascular procedures. As in other studies, follow up interventions were often repeated endovascular procedures [12,19,20,21]. Depending on stent localization and length, the puncture level can be selected proximal or distal to the stent. Puncture through the stent struts in particular of self-expanding stents is easily possible. It is also feasible to use an occlusion system through an implanted stent. 

## 5. Conclusions

Stent angioplasty of the CFA is a treatment option associated with low TLR rates. Peri-procedural complications can be treated conservatively or endovascularly in the majority of cases. Further comparative studies are needed to compare this endovascular option with surgical therapy in the long term, in particular for identifying potential lesion characteristics that may benefit from one or the other revascularization technique.

## Figures and Tables

**Table 1:** Baseline Characteristics.

Baseline Characteristics	*n* = 250	Mean ± SD (Range)
Age, years	70.8 ± 9.3	
Male sex	182 (72.8)	
Hypertension	223 (89.2)	
Diabetes mellitus	87 (34.8)	HbA1c 6.1 ± 0.99% (4.5–11.3%)
Hyperlipidemia	201 (80.4)	
		Triglyceride 169 ± 106 mg/dL (37–716)
		Total cholesterol 178 ± 45 mg/dL (86–377)
		HDL-cholesterol 53 ± 23 mg/dL (19–299)
		LDL-cholesterol 108 ± 40 mg/dL (26–317)
Current Smoker	94 (37.6)	
Former Smoker	94 (37.6)	
Coronary heart disease	111 (44.4)	
Cerebral vascular disease	61 (24.4)	
Renal insufficiency *	71 (28.4)	
Requiring dialysis	13 (5.2)	
Claudication	171 (68.4)	
Critical limb-threatening ischemia	79 (31.6)	
Rutherford-Becker class		
1	2 (0.8)	
2	42 (16.8)	
3	126 (50.4)	
4	38 (15.2)	
5	41 (16.4)	

Values are *n* (%). * defined as clearance < 60 mL/min.

**Table 2:** Lesion classification according to Rabellino et al. [11].

	I	II	III	IV
S (Stenosis)	63	65	40	17
O (Occlusion	26	8	12	19
H (Heavy)	51	54	41	26
M (Mild-moderate)	38	19	11	10
S (SFA)			12	4
D (DFA)			5	4
B (Both)			35	28

DFA—deep femoral artery, SFA—superficial femoral artery.

**Table 3:** Lesion and Index Procedure Characteristics.

	*n* (%)
Lesion Description	
De Novo	180 (72.0)
Reintervention (after endovascular treatment)	53 (21.2)
POBA	23
Cutting	1
DCB	12
Atherectomy/Thrombectomy + POBA or DCB	17
Reintervention (after surgical treatment)	11 (4.4)
Reintervention (after endovascular and surgical treatment)	6 (2.4)
Adjunctive target lesion therapy	
Plain old balloon angioplasty	234 (93.6)
Drug coated balloon angioplasty	64 (25.6)
Atherectomy	19 (7.6)
Non-target lesion interventions	
Femoral ipsilateral	166 (66.4)
Infrapopliteal ipsilateral	32 (12.8)
Contralateral	60 (24.0)

Values are *n* (%).

**Table 4:** Stent Characteristics.

	*n* (%)
Type of Stent	
Supera	31 (12.4)
Viabahn	2 (0.8)
Zilver PTX	9 (3.6)
Scuba	4 (1.6)
Smart	110 (44.0)
Lifestent	13 (5.2)
Absolute	35 (14.0)
Others	45 (18.0)
Number of Stents	1.06 ± 0.32
Length of Stents	46.4 ± 35.4
Diameter of Stents	7.7 ± 1.21

Values are *n* (%) or mean ± SD.

**Table 5:** Procedural Complications.

Procedural Complications	*n* = 23
Access site pseudoaneurysm	9 (3.6)
Occlusion/Stenosis due to closure system	2 (0.8)
Perforation/Bleeding (non-target lesion)	7 (2.8)
Distal Embolization	2 (0.8)
Compartment Syndrome	2 (0.8)
Technical Complications	1 (0.4)

**Table 6:** Clinical and Procedural Outcome.

Procedural Outcome	
Degree of Stenosis at Baseline	86.5 ± 10.7
Residual Stenosis ≤ 30%	236 (94.4)
Ankle–Brachial-Index	
Baseline	0.43 ± 0.35
Post-procedure	0.78 ± 0.38 (*p* < 0.001)
Follow up 6 months	0.76 ± 0.42 (*p* < 0.001)
Follow up 12 months	0.84 ± 0.41 (*p* < 0.001)
Follow up 24 months	0.74 ± 0.41 (*p* < 0.001)
Mean Rutherford–Becker Class	
Baseline	3.3 ± 0.98
Follow up 6 months	1.6 ± 1.63 (*p* < 0.001)
Follow up 12 months	1.8 ± 1.70 (*p* < 0.001)
Follow up 24 months	2.0 ± 1.61 (*p* < 0.001)
Target lesion revascularization	
Within 12 months	26 (10.4)
Within 24 months	41 (16.4)
Endovascular reintervention	30 (73.2)
Open, surgical treatment	11 (26.8)
Time to TLR (in months)	26.76 ± 7.7
Major amputation	4 (1.6)
Minor amputation	3 (1.2)

Values are *n* (%) or mean ± SD. TLR—target lesion revascularization.

**Table 7:** Predictors of TLR.

		Univariate Analysis			Mulivariate Analysis	
	HR	95%-CI	*p*-Value	HR	95%-CI	*p*-Value
Age (per year)	0.982	0.948–1.018	0.322			
Sex	1.661	0.725–3.804	0.230			
Hypertension	0.847	0.301–2.383	0.753			
Hyperlipidemia	1.920	0.711–5.181	0.198			
Diabetes mellitus	1.098	0.547–2.203	0.793			
Current Smoker	4.971	1.631–15.149	0.005	4.924	1.598–15.175	0.006
Coronary heart disease	2.529	1.265–5.056	0.009	2.626	1.285–5.365	0.008
Cerebral vascular disease	1.787	0.868–3.681	0.115			
Stroke	1.100	0.301–4.013	0.886			
Renal insufficiency	1.096	0.625–1.920	0.750			
CLTI	1.899	0.957–3.767	0.067			
Target lesion occlusion	1.279	0.628–2.606	0.498			
Severe calcification	0.739	0.154–3.779	0.739			
Residual stenosis	1.421	0.378–5.335	0.603			
POBA	1.400	0.306–6.407	0.665			
DCB	1.438	0.693–2.984	0.329			
Atherectomy	0.579	0.129–2.609	0.477			

95%-CI—95%-confidence interval for the Hazard ratio; CLTI—critical limb-threatening ischaemia; DCB—drug-coated balloon; HR—Hazard ratio; POBA—plain old balloon angioplasty.

## Data Availability

The data presented in this study are available in the article. The datasets generated during and/or analyzed during the current study are available from the corresponding author on reasonable request.
